# Correlation and Reliability of Behavioral and Otoacoustic-Emission Estimates of Contralateral Medial Olivocochlear Reflex Strength in Humans

**DOI:** 10.3389/fnins.2021.640127

**Published:** 2021-02-16

**Authors:** Miriam I. Marrufo-Pérez, Peter T. Johannesen, Enrique A. Lopez-Poveda

**Affiliations:** ^1^Instituto de Neurociencias de Castilla y León, Universidad de Salamanca, Salamanca, Spain; ^2^Instituto de Investigación Biomédica de Salamanca, Universidad de Salamanca, Salamanca, Spain; ^3^Departamento de Cirugía, Facultad de Medicina, Universidad de Salamanca, Salamanca, Spain

**Keywords:** basilar membrane, suppression, olivocochlear efferents, effective attenuation, input/output curves, contralateral acoustic stimulation

## Abstract

The roles of the medial olivocochlear reflex (MOCR) in human hearing have been widely investigated but remain controversial. We reason that this may be because the effects of MOCR activation on cochlear mechanical responses can be assessed only indirectly in healthy humans, and the different methods used to assess those effects possibly yield different and/or unreliable estimates. One aim of this study was to investigate the correlation between three methods often employed to assess the strength of MOCR activation by contralateral acoustic stimulation (CAS). We measured tone detection thresholds (*N* = 28), click-evoked otoacoustic emission (CEOAE) input/output (I/O) curves (*N* = 18), and distortion-product otoacoustic emission (DPOAE) I/O curves (*N* = 18) for various test frequencies in the presence and the absence of CAS (broadband noise of 60 dB SPL). As expected, CAS worsened tone detection thresholds, suppressed CEOAEs and DPOAEs, and horizontally shifted CEOAE and DPOAE I/O curves to higher levels. However, the CAS effect on tone detection thresholds was not correlated with the horizontal shift of CEOAE or DPOAE I/O curves, and the CAS-induced CEOAE suppression was not correlated with DPOAE suppression. Only the horizontal shifts of CEOAE and DPOAE I/O functions were correlated with each other at 1.5, 2, and 3 kHz. A second aim was to investigate which of the methods is more reliable. The test–retest variability of the CAS effect was high overall but smallest for tone detection thresholds and CEOAEs, suggesting that their use should be prioritized over the use of DPOAEs. Many factors not related with the MOCR, including the limited parametric space studied, the low resolution of the I/O curves, and the reduced numbers of observations due to data exclusion likely contributed to the weak correlations and the large test–retest variability noted. These findings can help us understand the inconsistencies among past studies and improve our understanding of the functional significance of the MOCR.

## Introduction

The central nervous system can adjust the functioning of the inner ear via the olivocochlear efferent system. Some efferent fibers originate in the medial region of the superior olivary complex and terminate on the outer hair cells (OHCs) in the cochlea (Warr and Guinan, 1979). These fibers, termed medial olivocochlear (MOC) efferents, can be activated reflexively by sounds presented to the ipsilateral and/or the contralateral ear (Liberman and Brown, 1986; Brown et al., 2003). It has been suggested that the MOC reflex (MOCR) serves to protect the auditory system from acoustic overstimulation and to facilitate auditory perception in noise, among other. However, the evidence in support of these roles is mixed (reviewed by Fuente, 2015; Smith and Keil, 2015; Lopez-Poveda, 2018). Because the effects of the MOCR can be assessed only indirectly in healthy humans, the existing evidence is mostly based on correlations between a psychoacoustic measure of interest (e.g., noise-induced temporary threshold shifts or speech-in-noise recognition) and indirect estimates of the inhibition of basilar membrane (BM) responses by MOCR activation, often referred to as MOCR strength. Different studies have used different techniques to estimate MOCR strength. If the different methods yielded uncorrelated or unreliable estimates of MOCR strength, this could partly explain the discrepant findings regarding the roles of the MOCR in human hearing. The aim of the present study was to investigate the correlation and reliability of three different methods often employed to estimate MOCR strength in humans.

Activation of MOC efferents hyperpolarizes OHCs (Cooper and Guinan, 2003), turning down the gain of the cochlear amplifier at low-to-mid levels and linearizing BM input/output (I/O) curves (Murugasu and Russell, 1996; Dolan et al., 1997; Cooper and Guinan, 2003, 2006; Guinan, 2006). For a tone in noise, MOC efferents inhibit the cochlear mechanical response to the noise and tone stimuli. As a result, auditory nerve fibers respond less to the background noise and show less ‘compressed’ rate-level functions (Winslow and Sachs, 1988; Kawase et al., 1993). Animal studies suggest that MOC efferents can protect the auditory system from acoustic trauma (Handrock and Zeisberg, 1982; Kujawa and Liberman, 1997; Maison and Liberman, 2000; Maison et al., 2013) and/or enhance the neural representation of transient stimuli in noisy backgrounds (Nieder and Nieder, 1970a, b). However, the results from human studies are not always consistent with these notions (Fuente, 2015; Lopez-Poveda, 2018).

In animals, the roles of MOC efferents have been studied by interrupting or sectioning the MOCR pathways (e.g., Handrock and Zeisberg, 1982; Warren and Liberman, 1989; Kujawa and Liberman, 1997; Maison et al., 2013). This approach is not always feasible in humans and vestibular neurectomy (the procedure employed to section olivocochlear efferents) is likely ineffective in cutting all olivocochlear efferents (Chays et al., 2003). For these reasons, many human studies have sought to establish a correlation between auditory perceptual tasks hypothesized to depend on the MOCR and an effect of MOCR activation on BM responses. Different methods have been used to assess MOCR effects. For example, many studies have estimated MOCR strength as the level change in click-evoked (CEOAEs) or distortion-product otoacoustic emissions (DPOAEs) induced by contralateral acoustic stimulation (CAS) (e.g., Giraud et al., 1997; Kumar and Vanaja, 2004; De Boer et al., 2012; Stuart and Butler, 2012; Abdala et al., 2014; Mishra and Lutman, 2014; Bidelman and Bhagat, 2015; Mertes et al., 2018, 2019). Because a contralateral broadband noise (BBN) with sufficient level [≥30 dB sound pressure level (SPL); Moulin et al., 1993] activates the contralateral MOCR, and because otoacoustic emissions (OAEs) require OHC-mediated amplification (Shera and Abdala, 2012), the suppression of CEOAEs or DPOAEs by CAS is thought to be the result of the MOCR reducing cochlear gain. MOCR strength has been also estimated as the CAS-induced change in OAE I/O curves (Moulin et al., 1993; Veuillet et al., 1996; Abdala et al., 1999), in behaviorally inferred BM I/O curves (Yasin et al., 2014; Fletcher et al., 2016), and in tone detection thresholds (Kawase et al., 2003; Aguilar et al., 2015; Nogueira et al., 2019).

It is yet to be shown, however, that the different methods used to assess MOCR strength in humans yield reliable and correlated results. In fact, studies aimed at investigating the facilitating role of the MOCR in speech-in-noise recognition have shown discrepant findings when using different methods to assess MOCR strength. For instance, monaural speech reception thresholds (SRTs) for sentences in noise are correlated with CAS-induced CEOAE suppression (Bidelman and Bhagat, 2015) but not with DPOAE suppression (Mukari and Mamat, 2008). Strikingly, findings can be discrepant even when MOCR strength is assessed using the same method. For example, Bidelman and Bhagat (2015) found SRTs for sentences in noise to be correlated with CEOAE suppression, while Stuart and Butler (2012) did not, something remarkable considering that the two studies measured CEOAE suppression using identical stimuli [60 dB peak-equivalent SPL (pSPL) linear clicks at a rate of 50/s and contralateral BBN of 65 SPL]. Mertes et al. (2018) observed a correlation between CAS-induced CEOAE suppression and the slope of the psychometric function for words in noise, but Mertes et al. (2019) did not find such a correlation for the same speech material. Notably, Mertes et al. (2018) measured CEOAEs using 75 dB pSPL clicks while Mertes et al. (2019) used 65 dB pSPL clicks. It is possible that differences across studies in the speech tests or participants contribute to the discrepant findings, but it is also possible that the effects of CAS on CEOAEs and DPOAEs are not reliable or equivalent to assess MOCR strength.

Here, we investigate the correlation and reliability of three popular ways of assessing the strength of the contralateral MOCR in humans. We measured pure-tone detection thresholds at different frequencies as well as CEOAEs and DPOAEs for different test frequencies and levels (i.e., I/O curves). All measures were obtained with and without CAS to compare the “CAS effect” across measures. They were also obtained multiple times to assess the variability of the CAS effect for each measure. Low test–retest variability together with a high correlation of the CAS effect between the different measures would support that the three measures are reliable and consistent, and thus serve equally to assess MOCR strength. By contrast, high test–retest variability and/or a lack of correlation between methods would indicate that different factors are probably involved in the CAS effects for each measure, which would help to understand the inconsistencies among studies and improve our understanding of the functional significance of the MOCR.

## Materials and Methods

### Participants

Twenty-eight subjects (21 women) with no self-reported history of hearing impairment participated in the study, although not all of them participated in every test (see below). Their mean age was 27.5 years (standard deviation, *SD* = 7.5 years; age range = 18–47 years). Air conduction audiometric thresholds were measured using a clinical audiometer (Interacoustics AD229e). All but three of the participants had air conduction audiometric thresholds ≤ 20 dB hearing level (HL) in both ears at frequencies between 125 Hz and 8 kHz (ANSI, 1996). The exceptions were two participants whose threshold was 25 dB HL at 8 kHz in the left and/or right ear, and another participant whose threshold was 60 dB HL at 8 kHz in the right ear. This latter participant was nevertheless admitted for testing because her thresholds were normal over the frequency range of interest for the present study (≤4 kHz). Twenty-six subjects had normal tympanograms (assessed using an Interacoustics AT235h clinical tympanometer and a test tone of 226 Hz at 85 dB SPL). Two listeners had slightly higher than typical values for ear-canal volume, compliance values, and/or tympanic peak pressure in one ear.

Participants were volunteers and not paid for their services.

### Tone Detection Thresholds

Absolute detection thresholds in the presence and in the absence of CAS were measured for tones presented monaurally in the left ear of 15 participants and in the right ear of 13 participants (*N* = 28 participants in total). Pure tone frequencies were 0.5, 1.5, and 4 kHz. The duration of the tones was 300 ms, including 10-ms raised-cosine onset and offset ramps. The CAS was a BBN (0.01–10 kHz). This noise bandwidth was used because it produces the greatest MOCR activation (Maison et al., 2000; Lilaonitkul and Guinan, 2009). The CAS level was 60 dB SPL. This level is capable of activating the MOCR with minimal or no activation of the middle-ear muscle reflex (Zhao and Dhar, 2010; Aguilar et al., 2013; Mishra and Lutman, 2013; Mertes and Leek, 2016; Feeney et al., 2017). The CAS had a duration of 850 ms, including 5-ms raised-cosine onset and offset ramps.

A three-interval, three-alternative, forced-choice adaptive procedure was used to measure tone detection thresholds. Three intervals were presented to the listener accompanied by brief lights in a computer monitor, and the tone was presented in one of the intervals chosen at random. The lights were on for 850 ms, and the inter-interval time (the period between the offset and the onset of the lights) was 500 ms. In the conditions with CAS, the CAS was presented in the three intervals gated with the lights. The tone started 500 ms after the light onset in the conditions with and without CAS. That is, the tone started 500 ms after the noise onset in the conditions with CAS. Because the MOCR is almost fully activated about 280 ms after the elicitor onset (Backus and Guinan, 2006), we assumed that the CAS-activated MOCR was fully active at the onset of the tone and remained active over the tone duration.

Participants were instructed to identify the interval containing the tone by pressing a key on the computer keyboard, and feedback was given on the correctness of their responses. The level of the tone decreased after two successive correct responses and increased after an incorrect response (two-down, one-up adaptive rule). The tone detection threshold was thus defined as the tone level giving 70.7% correct responses in the psychometric function (Levitt, 1971). The level of the tone changed by 6 dB until the second reversal in level occurred, and by 2 dB thereafter. The procedure continued until 12 level reversals occurred, and the detection threshold was defined as the mean of the tone levels at the last 10 reversals.

Tone thresholds with and without CAS were always measured in pairs without removing the earphones to avoid measurement variance from the earphones fit, and the threshold without CAS was always measured first. A given pair of thresholds was discarded when the within-measure SD for one or the two thresholds in the pair exceeded 4 dB. The exceptions were three participants for whom we accepted SD ≤ 6 dB at 0.5 kHz. Three threshold pairs (with and without CAS) were obtained for each tone frequency. When the across-measure SD of the three thresholds with or without CAS exceeded 4 dB, an additional pair of thresholds was measured. The three (or four) thresholds were averaged and the mean was taken as the tone detection threshold. Thresholds for the three test frequencies were measured in random order across participants.

### Click-Evoked Otoacoustic Emissions (CEOAEs)

Click-evoked otoacoustic emissions for the same ear as tone detection thresholds were measured in the presence and in the absence of CAS. CEAOEs were measured using the linear method, in which the responses to four clicks of the same amplitude and polarity were averaged (Kemp et al., 1990). This method was used because although the non-linear method is less sensitive to artifacts, it also cancels linear components of the OAEs and can eliminate much OAEs from the recording (Shera and Abdala, 2012), including the linear part of the MOC effect (Guinan, 2006). For each CEOAE measurement, 1,024 clicks of 75 μs in duration were presented at a rate of 19 Hz. The use of click rates ≤25 Hz minimizes the probability of clicks activating the ipsilateral MOCR (Boothalingam and Purcell, 2015). A 19.5 ms response window was used to extract the CEOAE level from the average waveform. The window started 2.5 ms after the end of the click to minimize stimulus artifact. In addition to the overall CEOAE level, the spectrum of the recording was calculated to obtain CEOAE levels at five frequency bands centered at 1, 1.5, 2, 3, and 4 kHz.

Click-evoked otoacoustic emissions for click levels of 51, 54, 57, and 60 dB pSPL^1^ were measured in 18, 28, 18, and 20 participants, respectively. In other words, full CEOAE I/O functions (i.e., CEOAEs for the four click levels) were obtained in 18 participants. Eight CEAOE measures (of 1,024 clicks each) were obtained for each click level. Four measures were obtained without CAS and four measures were obtained with CAS. Measurements with and without CAS were interleaved. For any given click level and frequency band, the mean CEOAE level with or without CAS was calculated when at least three of the four pair of measures (with and without CAS) were valid and when the across-measures SD was ≤3 dB both with and without CAS. A measure was regarded as valid when the signal-to-noise ratio (SNR) was ≥6 dB. The mean CEOAE level must be at least 3 dB higher than the system’s artifact level to be included in the analyses. If a measure did not meet these criteria, it was classified as “no response.”

The CAS had the same characteristics as described for tone detection thresholds, with the exception of its duration. Here, the CAS onset and offset were controlled manually by the experimenter. The CAS started one-to-two seconds before the presentation of the first click and was continuously on until one-to-two seconds after the presentation of the last click.

### Distortion-Product Otoacoustic Emissions (DPOAEs)

For 18 participants, 2*f*_1_ − *f*_2_ DPOAEs were measured in the same ear as tone detection thresholds in the presence and the absence of CAS. The primary *f*_2_ frequencies were 1, 1.5, 2, 3, and 4 kHz, and the *f*_2_/*f*_1_ ratio was fixed at 1.2. The level of primary tone *f*_2_ (*L*_2_) ranged from 30 to 50 dB SPL in 5-dB steps, and the level of primary tone *f*_1_ was set equal to *L*_1_ = 0.4*L*_2_ + 39, the rule proposed by Kummer et al. (1998) to obtain largest DPOAEs for *L*_2_ ≤ 65 dB SPL. The duration of the primary tones was 225 ms, and the inter-tone duration was 42 ms. A DPOAE measure for a given *f*_2_ and *L*_2_ combination included 10 stimulus trials. Eight DPOAE measures (of 10 trials each) were obtained for each *f*_2_ and *L*_2_ combination, i.e., four measures were obtained with CAS and four measures were obtained without CAS in interleaved order. The criteria used to calculate the mean DPOAE level across measures were the same as for CEOAEs.

The 2*f*_1_ − *f*_2_ DPOAE recorded in the ear canal is the vector sum of an OAE distortion component generated at the cochlear region tuned around the *f*_2_ primary tone and an OAE reflection component generated at the 2*f*_1_ − *f*_2_ cochlear region (Shera and Guinan, 1999; Talmadge et al., 1999; Kalluri and Shera, 2001; Shera and Abdala, 2012). CAS can affect the distortion and reflection components differently, and thus cause DPOAE levels to be sometimes greater in the CAS than in the control condition (Abdala et al., 2009; Deeter et al., 2009; Henin et al., 2011). For this reason, a suppressor tone near the 2*f*_1_ − *f*_2_ frequency was used in an attempt to suppress the reflection-source contribution to DPOAE (Heitmann et al., 1998; Talmadge et al., 1999; Kalluri and Shera, 2001; Konrad-Martin et al., 2001; Johnson et al., 2006). The suppressor frequency was 64, 59, 54, 54, and 54 Hz below 2*f*_1_ − *f*_2_ when *f*_2_ was 1, 1.5, 2, 3, or 4 kHz, respectively. The levels of the suppressor (*L*_3_) were calculated according to Figure 8 of Johnson et al. (2006). However, because Johnson et al. (2006) observed variability of up to 15 dB in the optimal suppressor level across participants, we decided to use a suppressor level 10 dB below the level determined by the linear fit for their group data. We made that decision in an attempt not to affect the distortion-source component for subjects who needed a lower suppressor level than the mean to remove the reflection-source component contribution. We used the data centered at 2 kHz from Johnson et al. (2006) to calculate the suppressor levels for *f*_2_ = 1, 1.5, and 2 kHz, and the data centered at 4 kHz to calculate the suppressor levels for *f*_2_ = 3 and 4 kHz.

The CAS had the same characteristics as described for tone detection thresholds with the exception of the duration. Here, the CAS onset and offset was controlled manually by the experimenter, as for CEOAEs.

### Apparatus

Pure tones and CAS were generated with custom-made Matlab software and played via an RME Fireface 400 soundcard at a sampling rate of 44.1 kHz, and with 24-bit resolution. Stimuli were presented to the participants using Etymotic ER-2 insert earphones. These earphones are designed to give a flat frequency response at the eardrum and have a nominal inter-aural attenuation of 70 dB that minimizes cross hearing. Stimuli were calibrated by coupling the earphones to a sound level meter (Brüel and Kjaer 2238) through a Zwislocki coupler (Knowles DB-100). Calibration was performed at 1 kHz and the measured sensitivity was applied to all frequencies.

CEOAE and DPOAE were measured using an Intelligent Hearing Systems Smart device (with SmartOAE software version 5.10) equipped with an Etymotic ER-10D probe. CEOAE stimuli were calibrated with a Zwislocki coupler (Knowles DB-100) by measuring peak intensity with a sound level meter (Brüel and Kjaer 2238). DPOAE stimuli were calibrated with the same Zwislocki coupler for each primary frequency (*f*_1_ and *f*_2_). In-the-ear pressure calibration was not performed. The system artifact was assessed by presenting clicks at different levels (CEOAEs) and different combinations of primary frequencies and levels (DPOAEs) to a microphone connected to the coupler.

Participants sat in a double-wall sound attenuating booth during all measurements. For tone detection thresholds, earphones were removed between each pair of measurements with and without CAS. Threshold pairs for a given probe frequency could be measured in the same or in different sessions, depending on the availability of the participant. The time lapse between sessions ranged from minutes to a few days (2.2 days on average). During OAE measurements, participants were asked to remain as steady as possible. The OAE probe remained in the participant’s ear throughout the whole OAE measurement session to minimize measurement variance from altering the position of the probe in the ear canal. During OAE measurements, we did not control if participants were attending to the stimulus.

### Quantification of CAS Effects

**FIGURE 1:**
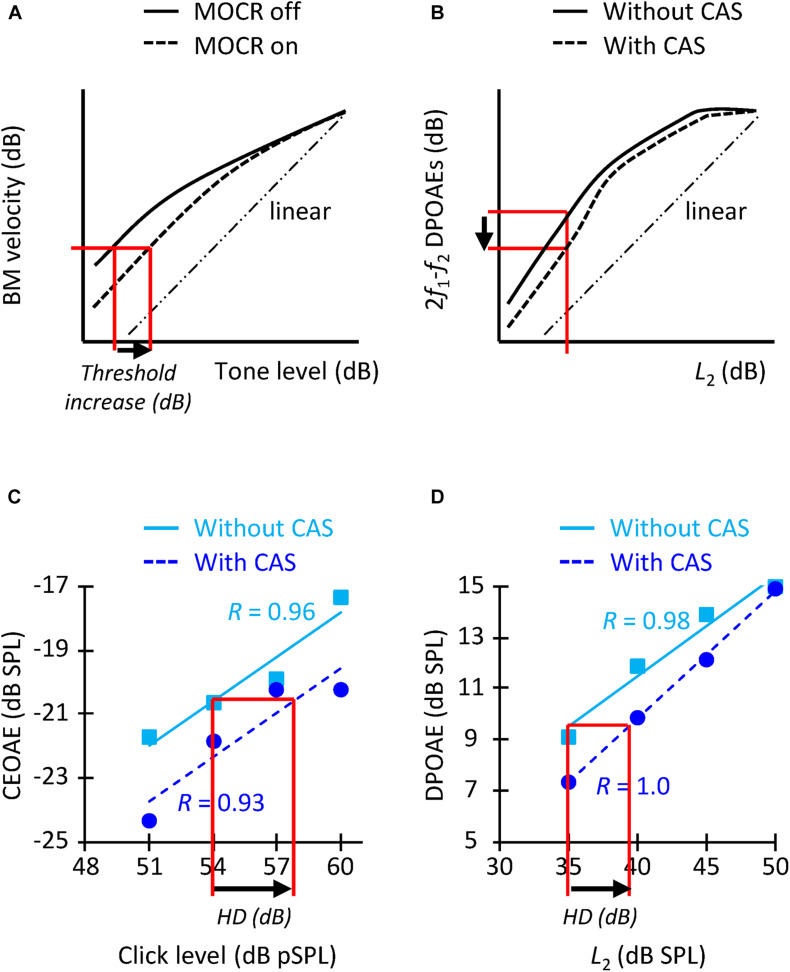
**(A)** Schematic representation of how MOCR activation is expected to change BM I/O curves (adapted from Cooper and Guinan, 2006) and increase tone detection thresholds (arrow). **(B)** Representation of how MOCR activation is expected to change DPOAE I/O curves [adapted from Moulin et al. (1993)] and reduce the DPOAE level for a given *L*_2_ level (arrow). **(C)** CEOAE I/O functions without (squares) and with (circles) CAS for an example participant. HD indicates the horizontal displacement (in dB). *R-*values indicate the correlation between the data and the linear fit. The I/O functions are for the frequency band of 1.5 kHz. **(D)** DPOAE I/O functions for *f*_2_ = 1.5 kHz for the same participant as in panel **(C)**. The layout is similar as for panel **(C)**.

Contralateral acoustic stimulation was expected to activate the contralateral MOCR, and thus to linearize BM I/O curves by inhibiting the gain of the BM at low-to-moderate levels (Figure 1A). Assuming that the BM response at the tone detection threshold is the same with and without CAS, we expected tone detection thresholds to be higher (worse) with than without CAS (Figure 1A). Because DPOAEs and CEOAEs require OHC-mediated amplification and CAS reduces such amplification, we also expected CAS to suppress CEOAEs and DPOAEs (Figure 1B). We quantified the CAS effect as the difference (in dB)^2^ in tone threshold, CEOAE level and DPOAE level in the CAS minus the control (no-CAS) condition, such that a positive threshold difference or a negative OAE difference would be consistent with BM inhibition/linearization.

It would not be appropriate, however, to compare the increase in tone detection threshold with the suppression of OAE levels at any one click level or *L*_2_ because the former presumably quantifies the horizontal displacement of BM I/O curve (also termed “effective attenuation”; Puria et al., 1996; Lichtenhan et al., 2016) (Figure 1A) while the latter probably quantifies the vertical displacement of the curve (Figure 1B). (Note that the horizontal and vertical displacements of the BM I/O curve are different when responses fall within the compressive region of the I/O curve). For this reason, we also quantified the CAS effect as the horizontal displacement of the CEOAE and DPOAE I/O curves. To do it, we first fitted straight lines to the data without and with CAS (Figures 1C,D). The fitting was done only when CEOAEs were present for at least two of the four click levels and when DPOAEs were present for at least three of the five *L*_2_ levels. The correlation between the fit and the data was ≥0.90 for 86% of I/O curves with more than three data points, which shows that the choice of a linear fit was appropriate. An I/O curve was excluded from the analyses when the correlation of the fit was <0.75 in the condition with or without CAS (7% of the cases). The horizontal displacement of CEOAE I/O curves was then calculated by estimating the CEOAE level in the fitted line without CAS produced by a click of 54 dB pSPL, followed by the click level in the CAS-fitted function that produced that same CEOAE level. The horizontal displacement was the difference between this latter value and 54 dB pSPL (arrow in Figure 1C). The CEOAE level without CAS for 54 dB pSPL clicks was obtained by extrapolation when the subject had valid CEOAE responses for two higher click levels. The same procedure was applied to estimate the horizontal displacement of DPOAE I/O curves, except that the displacement was calculated relative to the DPOAE responses for *L*_2_ = 35 dB SPL (arrow in Figure 1D). We calculated the shifts relative to 54 dB pSPL clicks and *L*_2_ = 35 dB SPL because very few participants had OAEs at lower click and *L*_2_ levels (Table 1).

**TABLE 1:** Statistical significance of the CAS effect for the different conditions of the study.

			CEOAEs				DPOAEs				
Freq. (kHz)		Tone thresholds	Click = 51 dB pSPL	Click = 54 dB pSPL	Click = 57 dB pSPL	Click = 60 dB pSPL	*L*_2_ = 30 dB SPL	*L*_2_ = 35 dB SPL	*L*_2_ = 40 dB SPL	*L*_2_ = 45 dB SPL	*L*_2_ = 50 dB SPL
Overall	*N*		5	12	8	8					
	*p*		1.000^†^	1.000^†^	0.558^†^	1.000					
0.5	*N*	28									
	*p*	**<0.001**									
1	*N*		7	17	15	15	4	8	11	12	11
	*p*		0.395	0.114^†^	0.095	**0.019**	1.000	0.345^†^	1.000^†^	0.115^†^	0.147
1.5	*N*	28	7	18	16	17	7	14	14	15	15
	*p*	**<0.001**	0.546^†^	**0.005^†^**	0.066^†^	**0.004**	0.161	**<0.001**	**<0.001**	**0.008**	**0.003**
2	*N*		5	13	13	15	6	7	10	14	13
	*p*		1.000	0.652	0.855	**0.024^†^**	1.000	0.854	0.062	**0.010^†^**	1.000
3	*N*		10	21	16	17	5	6	12	13	14
	*p*		**0.045**	**0.005^†^**	**0.002**	**0.003**	1.000	0.375^†^	0.266	1.000	0.769
4	*N*	28	7	11	10	13	7	10	12	13	15
	*p*	**<0.001**	**0.007**	**0.042**	**0.001**	**0.003**	1.000	0.305	1.000	0.664	**0.028**

**FIGURE 2:**
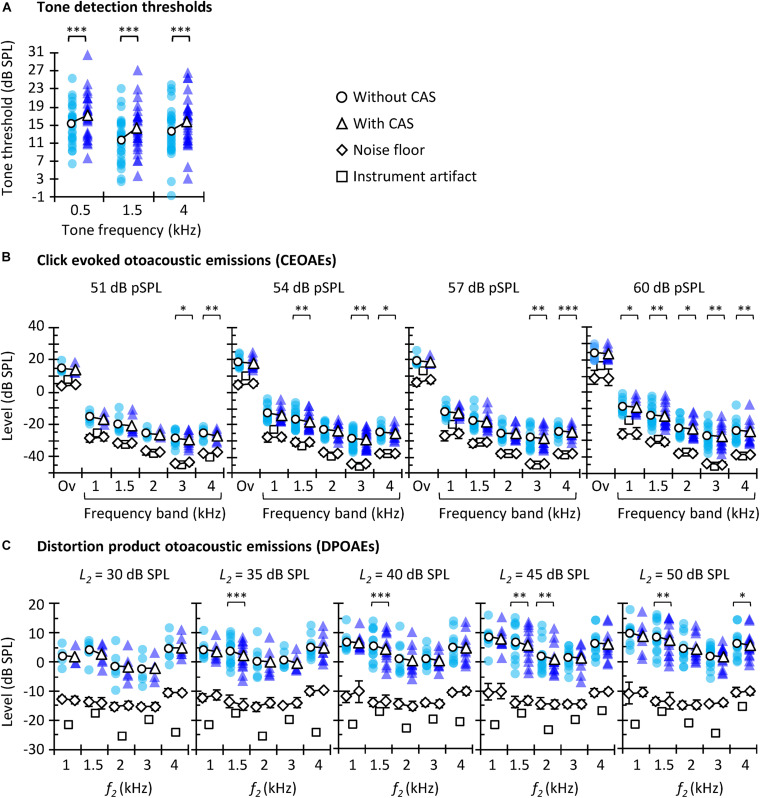
Tone detection thresholds **(A)**, CEOAEs **(B)**, and DPOAEs **(C)** without (circles) and with (triangles) CAS. Open symbols depict mean data and filled symbols depict individual results. For CEOAEs, scores are shown for different click levels and frequency bands, as well as for the overall (“Ov”) response, as indicated in each panel. For DPOAEs, scores are shown for the different *L*_2_ and *f*_2_. The background noise level (±one SD) is illustrated by diamonds, both without CAS (diamonds underneath the circles) and with CAS (diamonds underneath the triangles). The instrument artifact level is illustrated by squares. The exact number of participants for each probe frequency and level as well as exact *p*-values are shown in Table 1. Asterisks indicate statistically significant Bonferroni-corrected pairwise comparisons at ^∗^*p* ≤ 0.05, ^∗∗^*p* ≤ 0.01, ^∗∗∗^*p* ≤ 0.001.

*For a given condition, statistical significance was tested with non-parametric Wilcoxon signed rank test (^†^) or with paired t-test, as appropriate. p-values are corrected for multiple comparisons. Statistically significant effects (p ≤ 0.05) are highlighted using bold font. Note that the number of CEOAE and DPOAE responses used in the tests (N) is smaller than the number of participants (28 participants for tone thresholds and 54 dB pSPL clicks; 20 participants for 60 dB pSPL clicks; 18 participants for other conditions). This is because some measurements did not meet the inclusion criteria (see section “Materials and Methods”) and precluded us from using RMANOVAs or Friedman’s tests. Mean and individual results for each condition are depicted in Figure 2.*

### Quantification of the Reliability of the CAS Effect

The test–retest variability of the different estimates of MOCR strength was assessed in two ways. First, we correlated the magnitude of the CAS effect for trials #1, #2, and #3 and fitted a straight line to the data. If the measures were reliable, i.e., if CAS effect were equal across the three repetitions, the slope would be equal to 1.

The second analysis involved calculating the standard deviation of the CAS effect across trials #1, #2, #3, and/or #4. The more reliable measure would produce the smallest SD across trials. For tone detection thresholds, the SD of the CAS effect was calculated for the three measures at a given frequency. (Fourth measures were not included in the analysis because they were obtained only for some participants and frequencies; see above). For CEOAEs and DPOAEs, the CAS effect was calculated for the first, second, third, and fourth measures, and then the SD of the CAS effect across these measures was calculated. The SD of the CAS effect was calculated when at least three of the four pair of measures (with and without CAS) were valid. In this case, we did not request the across-measures SD to be ≤3 dB because enforcing that criterion would have reduced the actual SD.

### Statistical Analyses

**FIGURE 3:**
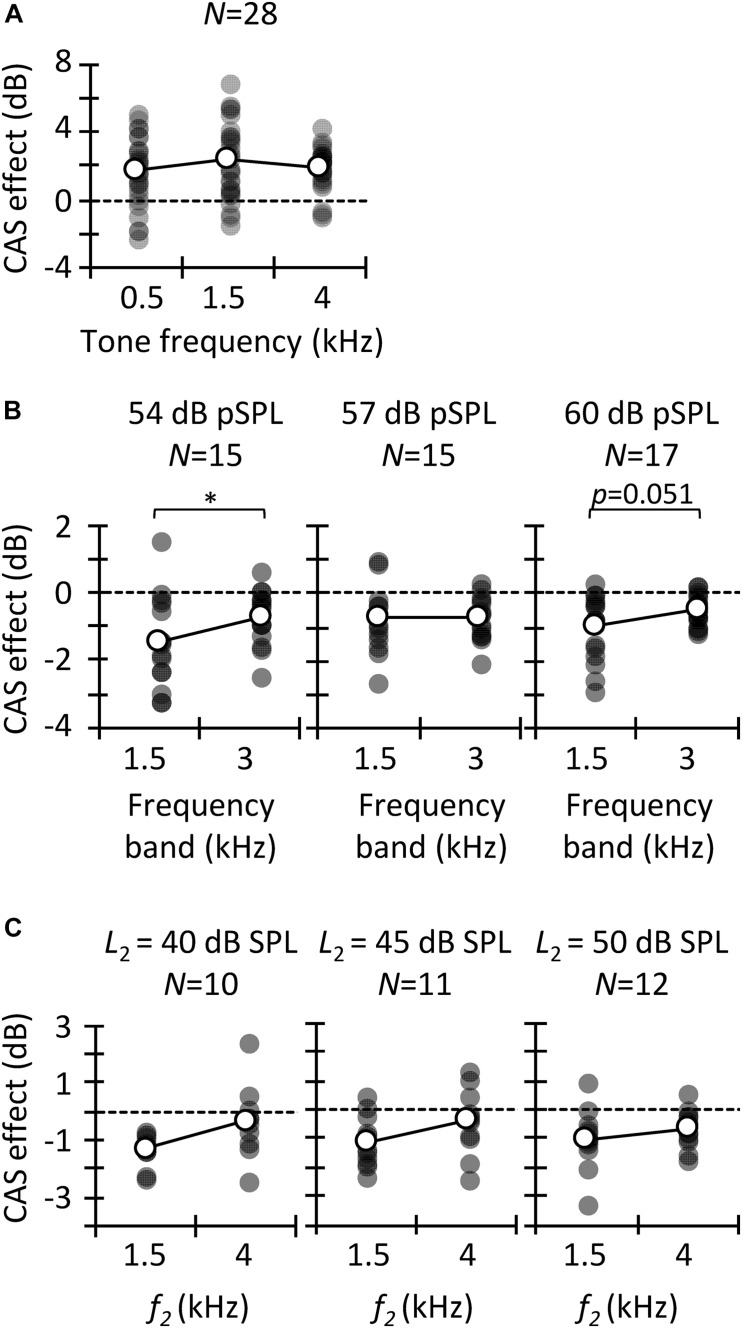
Contralateral acoustic stimulation (CAS) effect for different probe frequencies. Results are for tone detection thresholds **(A)**, CEOAEs **(B)**, and DPOAEs **(C)**. Open symbols depict mean data and filled symbols depict individual results. Data for a given click level or *L*_2_ are for the same participants, as indicated (*N*). Asterisks indicate statistically significant differences in pairwise comparison at *p* ≤ 0.05.

**FIGURE 4:**
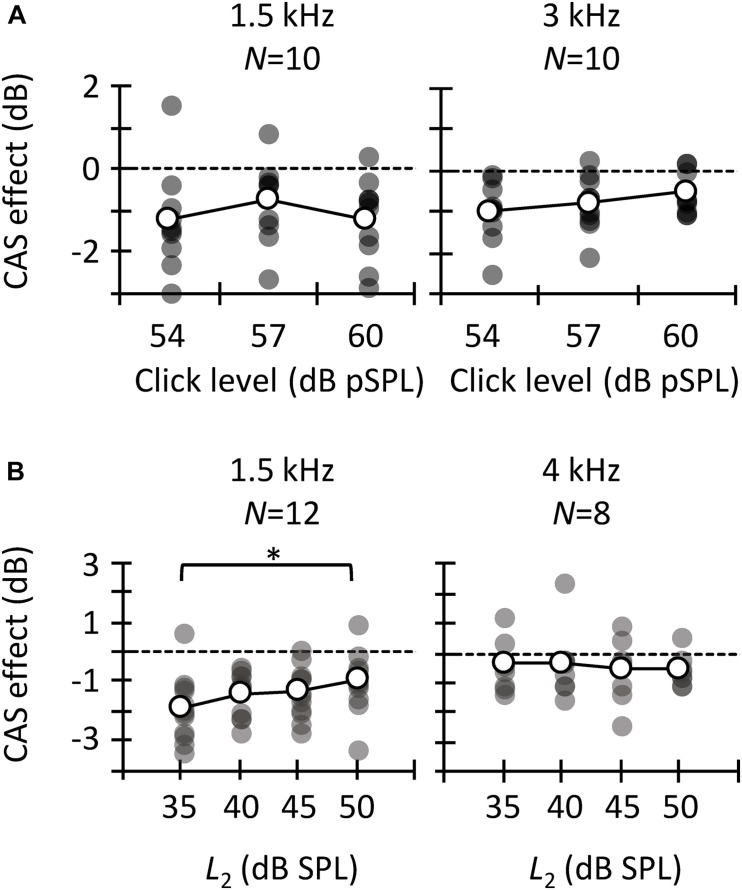
Contralateral acoustic stimulation (CAS) effect as a function of probe level. Results are for CEOAEs **(A)** and DPOAEs **(B)**. Data for a given frequency band **(A)** or *f*_2_ **(B)** are for the same participants (*N*). Open symbols depict mean data and filled symbols depict individual results. Asterisks indicate statistically significant differences in the pairwise comparison at *p* ≤ 0.05.

**FIGURE 5:**
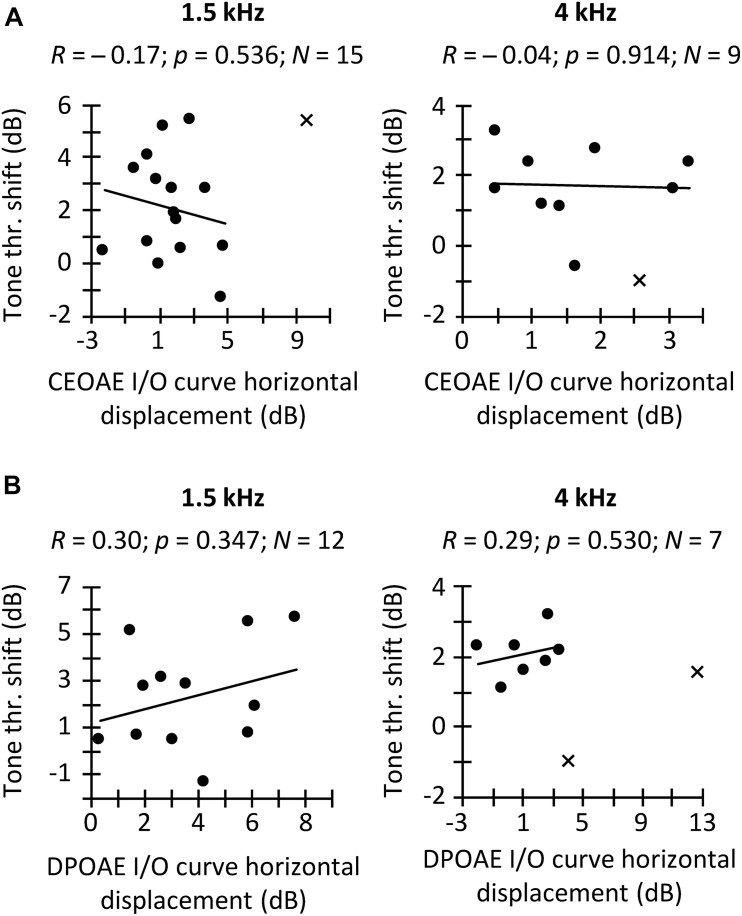
Correlation between the CAS effect on tone detection thresholds and on the horizontal displacement of CEOAE **(A)** and DPOAE **(B)** I/O curves. Separate panels are shown for frequency bands of 1.5 and 4 kHz **(A)** and for *f*_2_ of 1.5 and 4 kHz **(B)**, as indicated at the top of each panel. Data included in the correlation are depicted with circles, and outliers are depicted with crosses. A result was regarded as an outlier when it was outside 1.5 times the interquartile range. *R*-values indicate Pearson correlation coefficient. *p*-values were not corrected for multiple comparisons.

**FIGURE 6:**
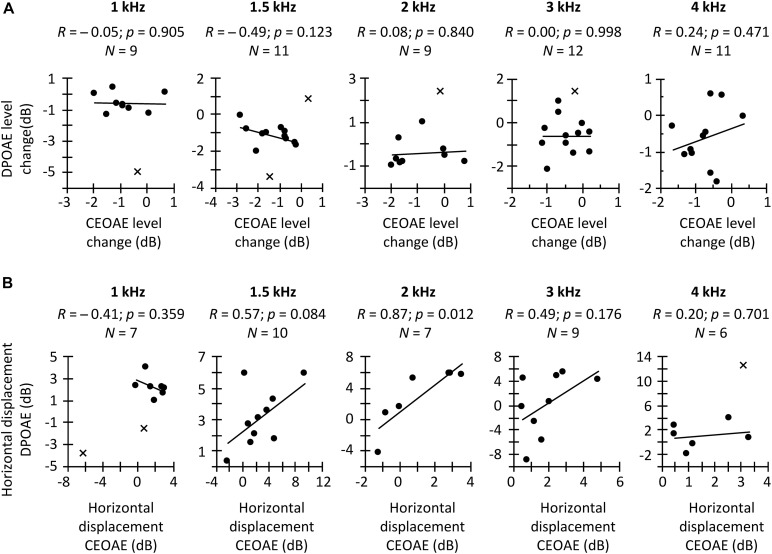
**(A)** Correlation between the CAS effect on CEOAEs for 60 dB pSPL clicks and on DPOAEs for *L*_2_ = 50 dB SPL. **(B)** Correlation between the CAS-induced horizontal displacement on CEOAE and DPOAE I/O curves. Correlations are for different frequency bands (CEOAE) or *f*_2_ (DPOAE), as indicated in each panel. Circles depict data points included in the correlation, while crosses depict outliers not included in the correlation. An outlier is not shown at 2-kHz in panel **(B)** because it was well outside the range of valid results. *R*-values indicate Pearson correlation coefficient. *p*-values were not corrected for multiple comparisons.

Statistical analyses were performed using IBM SPSS v. 23. Normality was tested with the Shapiro–Wilk test, and parametric or non-parametric tests were used as appropriate to evaluate the statistical significance of the CAS effect on tone detection thresholds, CEOAEs, and DPOAEs (Figure 2), as well as to evaluate the CAS effect for different probe frequencies (Figure 3) and levels (Figure 4). Pearson’s coefficient of correlation was used to investigate if there was a correlation between the different estimates of contralateral MOCR strength (Figures 5, 6). A score was regarded an outlier when it was outside 1.5 times the interquartile range. Outliers were not included in the correlations.

Because OAE data were not available for all participants and conditions (Table 1), the statistical tests used in the study focused on optimizing the analyses of the available data. For example, for any given click level, multiple *t* tests instead of a repeated-measures analysis of the variance (RMANOVA) were used to analyze the effect of CAS at every test frequency (Figure 2B); the RMANOVA would have excluded participants with missing data in some conditions. Similarly, for CEOAEs and DPOAEs, correlations were performed separately for each probe frequency and level instead of averaging data across all stimulus frequencies and/or levels, something that would have been interesting.

We applied two-tailed tests for all analyses. An effect was regarded as statistically significant when the null hypotheses could be rejected with 95% confidence (*p* ≤ 0.05). Unless otherwise stated, we applied Bonferroni corrections for multiple pairwise comparisons.

## Results and Discussion

### CAS Effect on Tone Detection Thresholds, CEOAEs, and DPOAEs

The aims of this study are to investigate (1) the correlation between three different methods often used to assess MOCR strength in humans; and (2) which of the three methods is more reliable. Before addressing these aims, however, we explored if the CAS had the expected effect of increasing tone thresholds and suppressing OAEs. Figure 2 shows tone detection thresholds (Figure 2A), CEOAE levels (Figure 2B), and DPOAE levels (Figure 2C) for all participants and test conditions. Note that there are fewer data points than participants were tested because some data did not meet the inclusion criteria (see section “Materials and Methods”). Average CEOAEs and DPOAEs were 13.9 and 16.6 dB, respectively, above the average noise floor (mean across probe levels and frequencies). These values indicate good quality of the OAEs recorded. Analyses showed that CAS increased tone detection thresholds and tended to suppress CEOAEs and DPOAEs, as expected. This trend occurred for all conditions, but the number of statistically significant pairwise comparisons was relatively greater at higher that at lower probe levels [i.e., for 54 and 60 dB pSPL click levels (Figure 2B) or *L*_2_ = 45 or 50 dB SPL (Figure 2C)] probably because of the larger number of data points at higher levels (Table 1).

### CAS Effect as a Function of Probe Frequency

We analyzed the CAS effect as a function of probe frequency to investigate to what extent our results are consistent across the three MOCR estimates as well as with previous studies. Figure 3A depicts the CAS effect on tone detection thresholds. The mean (±SD) magnitude of the CAS effect was 1.7 (±2.0), 2.3 (±2.2), and 2.0 (±1.2) dB for 0.5, 1.5, and 4 kHz, respectively. Friedman’s test did not reveal significant differences in the magnitude of CAS effect across frequency [χ^2^(2) = 1.5, *p* = 0.472]. This result is consistent with Aguilar et al. (2015) and Nogueira et al. (2019), who did not find significant differences in the effect of CAS on detection thresholds for 0.5- and 4-kHz tones when the duration of the tones was ≥200 ms. By contrast, Kawase et al. (2003) found greater CAS effect at 2 kHz than at lower or higher frequencies. On the other hand, the magnitude of the present CAS effect is comparable to that reported elsewhere (Aguilar et al., 2015; but see Kawase et al., 2003).

The magnitude of CAS effect on the 1.5- and 3-kHz components of CEOAEs is depicted in Figure 3B. Results are shown only at the two frequencies with the largest number of participants (Table 1). For 54 dB pSPL clicks, CEOAE suppression was greater at 1.5 than at 3 kHz (−1.4 vs. −0.7 dB) [*t*(14) = −2.25; *p* = 0.041]. For 57 dB pSPL clicks, CEOAE suppression was similar at 1.5 and 3 kHz (−0.75 vs. −0.72) [*t*(14) = −0.10; *p* = 0.921]. For 60 dB pSPL clicks, CEOAE suppression tended to be greater at 1.5 than at 3 kHz (−1.0 vs. −0.5 dB) [*t*(16) = −2.11; *p* = 0.051]. Our results are consistent with previous studies that found greater CAS effect on CEOAEs for frequency bands centered at or around 1.5 kHz than at 3 kHz (Francis and Guinan, 2010; Lisowska et al., 2014). In addition, the magnitude of CEOAE level suppression is in line with previous studies. For example, Francis and Guinan (2010) used 50 dB pSPL clicks and a contralateral BBN of 60 dB SPL and found CEOAE suppression about −1.5 dB for frequency bands ≤ 2.75 kHz, and about −0.5 dB for frequency bands between 3.25 and 5.25 kHz. Those values are close to the present estimates for 54 dB pSPL clicks, the closest level.

The magnitude of CAS effect on DPOAEs is depicted in Figure 3C for test frequencies of 1.5 and 4 kHz. Paired *t*-tests did not reveal a significant probe frequency effect when *L*_2_ was 40 dB SPL [*t*(9) = −1.98; *p* = 0.079], 45 dB SPL [*t*(10) = −1.54; *p* = 0.155], or 50 dB SPL [*t*(11) = −1.09 *p* = 0.300], although suppression tended to be greater at 1.5 than at 4 kHz. The mean suppression was −1.3 and −0.3 dB at 1.5 and 4 kHz, respectively, for *L*_2_ = 40 dB SPL; −1.4 and −0.4 dB, respectively, for *L*_2_ = 45 dB SPL; and −1.0 and −0.7, respectively, for *L*_2_ = 50 dB SPL. Previous studies have reported a gradual reduction of CAS effect with increasing primary frequencies (Wagner and Heyd, 2011; Abdala et al., 2014; Lisowska et al., 2014; Wicher and Moore, 2014), consistent with the present trend. The magnitude of DPOAE suppression is also consistent with previous studies. For example, Wicher and Moore (2014) reported an across-frequency mean DPOAE suppression of −1.4 (± 0.8) dB with contralateral BBN of 60 dB SPL and *L*_2_ = 50 dB SPL.

Altogether, the trend and magnitude of present CAS effects are consistent with those reported in previous studies. We found that the CAS effect on CEOAEs and DPOAEs tended to be greater at lower frequencies whereas it was fairly constant across frequencies for tone detection thresholds. This shows that the frequency dependence of the CAS effect was inconsistent for behavioral and OAEs measures.

### CAS Effect as a Function of Probe Level

Most physiological studies have shown that MOC activation suppresses BM responses (Murugasu and Russell, 1996; Dolan et al., 1997; Cooper and Guinan, 2006) and the compound action potential (Puria et al., 1996) more at lower than at higher levels, that is, over the range of stimulus levels where the cochlear amplifier gain is greatest (Robles and Ruggero, 2001). The CAS-induced suppression of CEOAEs is also usually greater at lower than at higher click levels (Hood et al., 1996; Veuillet et al., 1996; De Boer and Thornton, 2007; De Boer et al., 2012; Mishra and Lutman, 2013). Figure 4A shows the CAS effect on CEOAE levels as a function of click level for the 1.5 and 3 kHz frequency bands. The amount of CEOAE suppression was not significantly different for 54, 57, and 60 dB pSPL clicks neither at 1.5 kHz [one-way RMANOVA: *F*(2,18) = 1.46, *p* = 0.258] nor at 3 kHz [one-way RMANOVA: *F*(2,18) = 2.17, *p* = 0.143]. The absence of a level effect may be due to the narrow range of click levels studied. For example, Hood et al. (1996) found CEOAE suppression to be similar for 50, 55, and 60 dB pSPL clicks, and greater for those lower levels than for 65 or 70 dB pSPL clicks.

CAS-induced DPOAE suppression is also usually greater for lower than for higher primary levels (Moulin et al., 1993; Abdala et al., 1999; Wagner and Heyd, 2011). We found a statistically significant effect of primary level on DPOAE suppression at 1.5 kHz [one-way RMANOVA: *F*(3,33) = 2.91, *p* = 0.049] (Figure 4B). Pairwise comparisons with Bonferroni corrections showed greater DPOAE suppression for *L*_2_ = 35 dB SPL than for *L*_2_ = 50 dB SPL (*p* = 0.036). By contrast, we did not find a statistically significant effect of *L*_2_ level at 4 kHz [Friedman test: χ^2^(3) = 0.60, *p* = 0.896].

### Within-Subject Correlation of CAS Effect Across Methods

In a first analysis, we investigated the hypothesized within-subject correlation between CAS-induced increase in tone detection threshold and the horizontal displacement of CEOAE or DPOAE I/O curves. Results are shown in Figure 5. We found the expected trend only for DPOAEs, although the correlations were far from statistically significant (Figure 5B). The pattern of trends suggests that increasing the sample size might bring the correlation between threshold shifts and the horizontal displacement of DPOAE I/O curves closer to statistical significance but would unlikely reveal a correlation between threshold shifts and CEOAE I/O curve shifts. In other words, although the CAS-induced increase in tone detection thresholds and the horizontal displacement of CEOAEs I/O curves are both expected to be the result of the MOCR linearizing BM responses (Figure 1), those measures are not equivalent in revealing MOCR effects, at least when using the limited range of click levels used here. Our result agrees with Fletcher et al. (2016), who did not find a correlation between CEOAE suppression and the reduction of cochlear mechanical gain inferred from temporal masking curves.

In a second analysis, we investigated the hypothesized within-subject correlation between the CAS-induced suppression of CEOAEs and DPOAEs for CEOAEs and DPOAEs obtained at fixed, and roughly matched levels. BM responses to tones can be predicted from BM responses to clicks (Recio et al., 1998) but click and tone levels must be different to obtain the same BM response magnitude with the two stimuli. For example, in Recio et al. (1998), BM responses to 54-dB pSPL clicks predicted accurately the magnitudes of BM responses to 40-dB SPL tones in the chinchilla cochlea. Here, it is hard to know which click level and *L*_2_ produced the same BM response magnitude without CAS. For this reason, we opted to correlate the CAS effect for conditions with the greater number of data points. Figure 6A shows the within-subject correlation of the CAS effect on CEOAEs for 60 dB pSPL clicks and on DPOAEs for *L*_2_ = 50 dB SPL. The correlation was not significant at any probe frequency. Moreover, the expected trend occurred only at 4 kHz. Complementary analyses (not shown) revealed no significant correlations when using 60 dB pSPL clicks and *L*_2_ of 45 dB SPL, or 57 dB pSPL clicks and *L*_2_ of 50 or 45 dB SPL. The lack of correlation suggests that it is not appropriate to assume that the CAS effects on CEOAEs for a single click level and DPOAEs for a single *L*_2_ provide related information or can be used equivalently. It remains uncertain, however, whether associations would emerge across a broader parametric range (e.g., for other probe levels or averaging several probe levels).

In a third and last analysis, we investigated a potential within-subject correlation between the CAS-induced horizontal displacement of CEOAE and DPOAE I/O curves. Figure 6B shows that the expected trend occurred at intermediate frequencies (1.5, 2, and 3 kHz), and that the correlation was indeed statistically significant at 2 kHz. This suggests that the horizontal displacement of CEOAEs and DPOAEs I/O curves may be used somewhat ‘equivalently,’ at least at these frequencies. It remains uncertain, however, to what extent these displacements are reflecting MOCR effects. For instance, Lichtenhan et al. (2016) measured the CAS effect on the horizontal displacement of DPOAE I/O functions and on the compound action potential I/O functions in humans and found average trends to be discrepant (their Figure 4), which suggests that factors different from the MOCR are involved in one or both measures.

In summary, the CAS effect on tone detection thresholds was not correlated with the horizontal displacement of CEOAE and DPOAE I/O curves measured in the same subject. Similarly, for fixed stimulus levels, CAS-induced CEOAE suppression was not correlated with CAS-induced DPOAE suppression. The results also showed, however, that the horizontal displacements of CEOAE and DPOAE I/O curves were correlated with each other, at least for mid-frequency probes. The overall lack of correlation can be due to many factors, including the limited parametric space studied (e.g., the clicks and primary levels used here may represent different points in the CEOAE and DPOAE amplitude growth function), the limited resolution of I/O curves (e.g., the 9 dB range of click levels for CEOAE I/O curves may be too narrow to properly define the amplitude growth function), and/or the reduced numbers of observations due to data exclusion. However, other factors such as a low reliability of the measures could be another possible cause (see below).

### Reliability of CAS Effects

**FIGURE 7:**
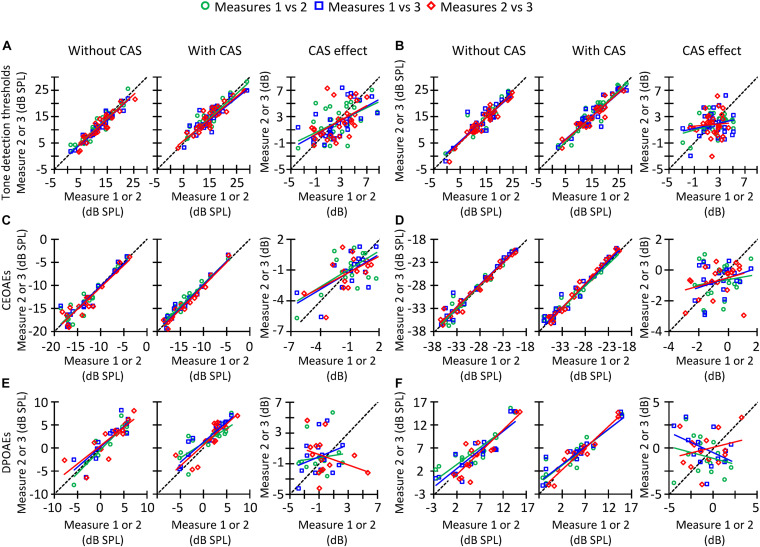
Correlation between trials #1 and #2 (circles), #1 and #3 (squares), or #2 and #3 (diamonds) of tone detection thresholds, CEOAEs, and DPOAEs in the conditions without CAS (left panels), with CAS (mid panels), as well as for the CAS effect (right panels). **(A)** Tone detection thresholds at 1.5 kHz. **(B)** Tone detection thresholds at 4 kHz. **(C)** CEOAEs for 54 dB pSPL clicks and for the frequency band of 1 kHz. **(D)** CEOAEs for 54 dB pSPL clicks and for the frequency band of 3 kHz. **(E)** DPOAEs for *L*_2_ = 50 dB SPL and *f*_2_ = 3 kHz. **(F)** DPOAEs for *L*_2_ = 50 dB SPL and *f*_2_ = 4 kHz. The dashed line indicates a 1-to-1 relation across trials, thus the points located on that line indicate highly reliable measures. Each data point represents the results for one participant.

In the preceding sections, it has been shown that the three different methods used to estimate the MOCR strength are not correlated with each other (Figures 5, 6). This can be partly due to the low reliability of the measures. Figure 7 illustrates across-trial correlations for tone detection thresholds (Figures 7A,B), CEOAEs (Figures 7C,D), and DPOAEs (Figures 7E,F) in the conditions without (left panels) and with CAS (mid panels), as well as for the CAS effect (right panels). In all panels, the dashed lines illustrate 1-to-1 test–retest correspondence, i.e., zero test–retest variability. For measures obtained with and without CAS, most symbols are located along the dashed line, indicating small test–retest variability. By contrast, for the CAS effect, symbols are away from the dashed line (right-most column in Figure 7), indicating high test–retest variability. This variability can be quantified by the slope of a linear fit to the data in each panel of Figure 7. For tone detection thresholds at 4 kHz (Figure 7B), the slope of the fitted function for measures #2 and #3 (red line) was 0.94 dB/dB in the condition without CAS, 0.88 dB/dB in the condition with CAS, and 0.10 dB/dB for the CAS effect. Because the slope was very different from 1 dB/dB in the latter case, we conclude that the CAS effect on 4-kHz tone detection thresholds is not reliable. Similar patterns are observed for CEOAEs (Figures 7C,D) and DPOAEs (Figures 7E,F), something surprising considering that OAE measures with and without CAS were obtained without removing the OAE probe from the participant’s ear. Altogether, the present results indicate that neither CAS-induced increases in tone threshold nor OAE suppression are reliable estimates of MOCR strength.

**FIGURE 8:**
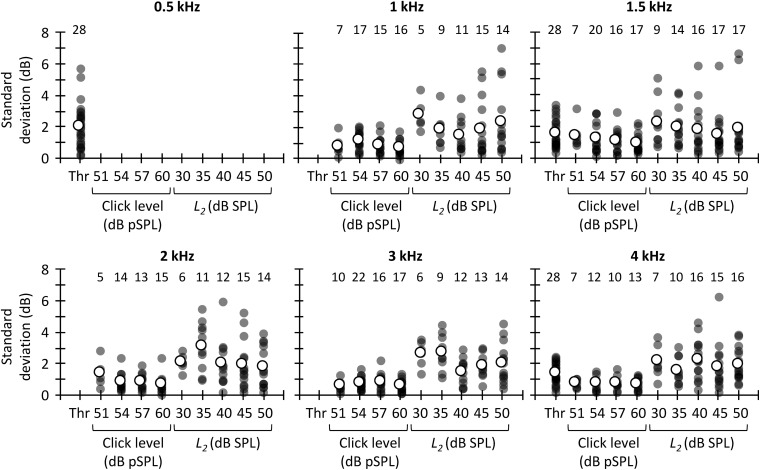
Across-measures standard deviation of CAS effect for tone detection thresholds, CEOAEs, and DPOAEs. Results are for different probe frequencies, click levels (CEOAEs) and *L*_2_ (DPOAEs). Open symbols depict mean data and filled symbols depict individual results. The numbers above each set of circles indicate the number of participants included in the analysis. One outlier for *L*_2_ = 30 dB SPL and *f*_2_ = 3 kHz is not shown in the figure and was omitted from the mean.

If one of the three methods considered here must be chosen to estimate MOCR strength, however, it would be useful to know which one is the most reliable to prioritize its use over the other one(s). Figure 8 shows the SD of the CAS effect across different trials for tone detection thresholds, CEOAEs and DPOAEs. At all frequencies, the SD was greater for DPOAEs than for tone detection thresholds or CEOAEs, demonstrating that CEOAEs or tone detection thresholds provide more reliable estimates of CAS effects than do DPOAEs. However, as noted earlier, reliability of the CAS effect on tone detection thresholds or CEOAEs can be also low at some frequencies (Figure 7).

The greater test–retest reliability of the CAS effect for CEOAEs than for DPOAEs is consistent with previous studies. Stuart and Cobb (2015) and Mertes and Goodman (2016) used Cronbach’s alpha, where a value of 1 indicates perfect reliability, to assess the intra-session test–retest reliability of CAS effect on CEOAES. Stuart and Cobb (2015) reported a Cronbach’s alpha greater than 0.8, and Mertes and Goodman (2016) reported a Cronbach’s alpha greater than 0.95. Kumar et al. (2012) and Kalaiah et al. (2018), however, reported a mean (across frequency) intra-session Cronbach’s alpha for DPOAEs of 0.5 and 0.3, respectively. It is unclear why reliability of the CAS effect was lower for DPOAEs than for CEOAEs. Kumar et al. (2012) and Kalaiah et al. (2018) proposed that attentional status might have contributed to the low test–retest reliability of DPOAEs. We, however, measured CEOAEs and DPOAEs in the same participants, and there is no reason to think that attentional status was more variable when measuring DPOAEs than CEOAEs. On the other hand, one might argue that the large test–retest variability for DPOAEs is related with the dual source (reflection and distortion) generation mechanism. DP-grams, however, are highly stable across measurement sessions (Gaskill and Brown, 1990; Zhang et al., 2007). That is, the amplitude and phase of the reflection and distortion component would not change (or not too much) from one trial to another. Hence, although possible, it is uncertain how the MOCR would affect differently the reflection and/or distortion components from one trial to another.

The present SDs of the CAS effect (Figure 8) are also in line with previous studies. Stuart and Cobb (2015) reported an intra-session SD of ∼0.4 dB when they used 60 dB pSPL clicks. We observed a mean (across frequency) SD of 0.7 dB for 60 dB pSPL clicks, the most similar condition. Kumar et al. (2012) quantified the variability of the CAS effect on DPOAEs as the standard error across-measures. They reported a mean (across frequency) standard error of 0.8 dB for *L*_2_ = 55 dB SPL. Here, the across frequency mean standard error was 1.0 dB for *L*_2_ = 50 dB SPL.

## General Discussion

We have shown that, on average, the use of a contralateral broadband noise increased tone detection thresholds, suppressed CEOAEs and DPOAEs, and horizontally shifted CEOAE and DPOAE I/O curves to higher levels, as expected. However, no correlations were found between the CAS effect on tone detection thresholds and on the horizontal displacement of CEOAEs or DPOAEs I/O curves (Figure 5), or between the CAS-induced suppression of CEOAEs at DPOAEs for a given stimulus level (Figure 6A). The horizontal displacements of CEOAE and DPOAE I/O curves were, however, correlated with each other, at least for the conditions with the greatest number of subjects (Figure 6B). We also found that the CAS effect on tone detection thresholds and CEOAEs showed the lowest test–retest variability, suggesting that their use should be prioritized over the use of DPOAEs.

### Possible Factors Responsible for the Lack of Correlation Across the Different Measures

The lack of correlation across the different MOCR strength estimates may be due to various factors. First, the restricted parameters employed could be a possible reason. We correlated CAS effects on CEOAEs and DPOAEs for fixed probe levels that may represent different points in the CEOAE and DPOAE amplitude growth function and thus result in weak or absent correlations. In addition, CEOAE and DPOAE I/O curves comprised only 2 or 3 points for some participants. This limited I/O curves resolution may have been insufficient to accurately define the amplitude growth. Further studies should test whether correlations emerge after exploring a broader parametric range.

Second, the CAS-induced increments in tone detection thresholds may reflect ‘central masking’ in addition to, or instead of, a linearization of BM responses by contralateral MOCR activation. That is, the CAS could have interacted with the test tone in the central auditory nervous system making tone detection harder, a phenomenon referred to as ‘central masking.’ Evidence in favor of central masking on tone detection thresholds has been reported previously. Smith et al. (2000) demonstrated that, in macaques, the tone threshold increment with CAS remained to some extent when MOC efferents were sectioned. Marrufo-Pérez et al. (2018) showed that detection thresholds for short (50 ms) tones increased more when the tone and CAS onset coincided (early condition) than when the tone onset was delayed 300 ms from the CAS onset (late condition). Because the time course of MOCR activation is around 300 ms (Backus and Guinan, 2006), one would expect greater threshold increments in the ‘late’ than in the ‘early’ condition if the MOCR were the only responsible for the increments, but this was not the case. In addition, several studies have demonstrated that bilateral cochlear implant users show an increase in the detection threshold of a probe signal in the presence of contralateral electric stimulation (Van Hoesel and Clark, 1997; James et al., 2001; Lin et al., 2013; Aronoff et al., 2015; Lee and Aronoff, 2018) despite the electrical stimulation delivered by cochlear implants bypasses OHCs and hence is independent from the MOCR. It is hard to differentiate the contribution of the MOCR from central masking. In addition, it is uncertain why some participants showed lower tone detection thresholds with than without CAS (Figure 3A), especially considering that both MOCR activation and central masking should have resulted in higher tone detection thresholds.

Third, the attentional state of the participants during the OAE measurements might have affected the results. Several studies have reported that auditory or visual selective attention can alter transient evoked OAEs (Froehlich et al., 1993a; De Boer and Thornton, 2007; Garinis et al., 2011; Namasivayam et al., 2015), DPOAEs (Smith et al., 2012; Srinivasan et al., 2012, 2014; Wittekindt et al., 2014), or the compound action potential (Delano et al., 2007), presumably by activation of the MOCR. In the present tone-detection experiment, participants must have attended to both visual (the lights displayed in the computer screen) and acoustic cues (the tones) during the measurements. CEOAEs and DPOAEs, by contrast, were recorded without controlling the attentional state of the participant. Therefore, it is uncertain if and to what extent participants were attending to the acoustic stimuli during OAE measurements. Moreover, some participants slept during OAE recordings, and sleeping can decrease efferent activity (Froehlich et al., 1993b). These factors could be partly responsible for the weak (or lack of) correlation between the CAS effect on tone detection thresholds, CEOAEs and/or DPOAES.

Fourth, the middle-ear muscle reflex (MEMR) could have confounded the results to some extent. We set the level of the contralateral BBN at 60 dB SPL because this level has been often used as MOCR elicitor (e.g., Abdala et al., 1999, 2009, 2014; Wagner et al., 2008; Francis and Guinan, 2010; Wicher and Moore, 2014; Aguilar et al., 2015; Boothalingam and Purcell, 2015; Mertes and Leek, 2016). Using the same level of CAS for all participants, however, may not be ideal because some listeners can have a MEMR threshold as low as 50 dB SPL for BBN (Zhao and Dhar, 2010; Feeney et al., 2017). The contraction of the middle-ear muscle changes middle-ear transmission and hence OAEs. If our contralateral stimulation activated the MEMR in some participants but not in others, this would introduce uncertainty and variability in the measures of MOCR strength. In addition, if the probability of MEMR activation was different for DPOAEs, CEOAEs, or threshold measurements, this could have contributed to the poor correlation and reliability of the measures.

Fifth, the lack of correlation between CAS-induced CEOAE and DPOAE suppression for a given stimulus level (i.e., for a given click level and *L*_2_) may have occurred to some uncertain extent because the third tone used when measuring DPOAEs did not suppress totally the reflection component. CAS changes the phase of the reflection component but not (or not so much) the phase of the distortion component, thus resulting in an increase of the DPOAE level when the two components change from canceling each other in the condition without CAS to combining in a constructive fashion in the condition with CAS (Deeter et al., 2009; Henin et al., 2011). As described in the Section “Materials and Methods,” the level of the suppressor tone was 10 dB below that suggested by Johnson et al. (2006). This level may have been insufficient to suppress the reflection-source contribution for some participants, which could explain why CAS sometimes enhanced rather suppressed DPOAEs (e.g., Figures 3C, 4B), thus resulting in CAS effect on DPOAEs to be an unreliable MOCR estimate.

Sixth, we did not control for the effects of standing waves in the ear canal, which can be present above 2–3 kHz and lead to inaccurate measurement of stimulus levels. Standing waves occurs when the stimulus presented to the ear canal (forward waveform) interacts with the stimulus reflected from the eardrum (backward waveform). These waveforms can enhance or cancel each other when are in phase or out of phase, respectively, resulting in a difference in the probe level between the microphone and eardrum of up to 20 dB (Stinson et al., 1982; Siegel, 1994). If standing waves were present during OAE recordings, they probably introduced noise into the measurements and consequently, the MOCR gauged by OAEs.

Seventh, as described previously, test–retest repeatability of the CAS effect for tone detection thresholds, CEOAES, and DPOAEs was very low for some probe frequencies (Figure 7) despite the various OAE trials (with and without CAS) were measured in a single session without removing the OAE probe. This low reliability can also contribute to the low (or lack of) correlation across the three methods used to estimate the MOCR strength. It is uncertain why the test–retest repeatability was low. One or more of the factors described in the preceding paragraphs (e.g., attentional status) could be responsible for it. Complementary Bland–Altman analyses (Bland and Altman, 1999) revealed that there was not a systematic bias of the measures from trial #1 to #3, i.e., the difference of the CAS effect between trials 1 and 3 was zero on average for tone detection thresholds, CEOAEs, and DPOAEs (results not shown). This indicates that the factor(s) that causes the low repeatability of the measures was independent of trial order.

Eighth, all participants were normal-hearers with presumably normal efferent system reflexes. It is possible that the natural scatter or variation in the MOCR strength was not large enough to capture a correlation well, if it exists.

Lastly, a potential problem is that CAS-induced changes in OAEs level need not reflect the reduction in the cochlear-amplifier gain, as is usually assumed. For example, Berezina-Greene and Guinan (2017) demonstrated that SFOAE amplitude increased at some frequencies and decreased at others when MOC efferents were activated by brainstem shocks in guinea pigs, and the increments occurred despite the animals showed a *reduction* in the cochlear-amplifier gain. Similar results might occur for CEOAEs insofar as CEOAEs and SFOAEs are generated by the same mechanism (Kalluri and Shera, 2007; Francis and Guinan, 2010; Shera and Abdala, 2012). Indeed, we found that CAS sometimes increased CEOAEs in some conditions (e.g., Figure 3A).

In summary, because the correspondence between the two OAE indices was explored across a limited (and maybe not always matching) range of stimulus levels and frequencies, and because many factors were not or could not be (e.g., central masking) controlled for, it is not surprising that correlations were not observed. Further research is needed to investigate which factors are mostly responsible for this lack of correlation and how their effects can be controlled for, as well as to design better measures of MOCR strength in humans.

### Implications

Current evidence supporting the roles of the MOCR in human hearing is mixed (reviewed by Fuente, 2015; Smith and Keil, 2015; Lopez-Poveda, 2018). The discrepant results across studies can be due to some extent to the methodology used. As in our study, many previous studies did not control for the attentional state of the participants (e.g., Kumar and Vanaja, 2004; Kim et al., 2006; Bidelman and Bhagat, 2015), the presence of fine structure in DPOAEs (e.g., Kim et al., 2006; Mukari and Mamat, 2008), or the presence of standing waves (e.g., Giraud et al., 1997; Kumar and Vanaja, 2004; Kim et al., 2006; Mukari and Mamat, 2008; Bidelman and Bhagat, 2015). In addition, many studies correlated a single estimate of the contralateral MOCR strength (e.g., CAS-induced suppression of CEOAEs for a single click level or DPOAEs for a single combination of primary levels *L*_1_ and *L*_2_) with performance scores in a psychoacoustical task of interest (e.g., Kumar and Vanaja, 2004; Kim et al., 2006; Mukari and Mamat, 2008; Stuart and Butler, 2012; Mishra and Lutman, 2014; Bidelman and Bhagat, 2015; Mertes et al., 2018, 2019). Here, we have shown that CEOAE suppression for a given click level is not correlated with DPOAE suppression for a given *L*_2_ (Figure 6A). Hence, it is not surprising that studies reached different conclusions about the roles of the MOCR in human hearing when the MOCR strength was estimated with two different methods and a single probe level [e.g., Mukari and Mamat (2008) and Bidelman and Bhagat (2015)]. On the other hand, some studies have measured DPOAE or CEOAE suppression by performing only one measure without CAS and another measure with CAS (e.g., Kumar and Vanaja, 2004; Stuart and Butler, 2012; Bidelman and Bhagat, 2015; Mertes et al., 2019). Here, we have shown that the suppression of CEOAEs and DPOAEs can be highly variable from trial to trial (Figures 7, 8). Therefore, it is also not surprising that findings were also discrepant across studies that aimed at investigating the roles of the MOCR in human hearing using the same methodology but assessing MOCR strength with only one measure without and with CAS [e.g., Stuart and Butler (2012) and Bidelman and Bhagat (2015)].

Altogether, our study suggests that many confound factors enter into MOCR measurement and that previous studies may have used a simplistic way of evaluating MOCR strength. How to optimize MOCR measurements must be addressed in further studies. In addition, other ways of analyzing OAEs could be explored. For example, Dragicevic et al. (2019) studied low-frequency (1–35 Hz) oscillatory amplitude changes in DPOAEs and electroencephalography to assess whether cortical oscillations modulate cochlear responses during selective attention. Their results were according to their hypothesis, and they propose the auditory efferent system as the most probable neural pathway responsible for modulating cochlear responses. It is possible that using such novel methods for OAE analyses help to investigate the roles of the MOCR in human hearing.

## Conclusion

(1) On average, contralateral acoustic stimulation (CAS) increased tone detection thresholds and decreased CEOAE and DPOAE levels in normal hearing listeners.
(2) The magnitude of CAS effect tended to be greater for lower (1.5 kHz) than for higher (3–4 kHz) frequencies for CEOAEs and DPOAEs. The effect of CAS on tone detection thresholds, however, was similar in magnitude for 0.5, 1.5, and 4 kHz probe tones.
(3) The CAS effect on CEOAEs was not different for 54, 57, and 60 dB pSPL clicks. The CAS effect on DPOAEs was greater for *L*_2_ = 35 dB SPL than for *L*_2_ = 50 dB SPL at 1.5 but not at 4 kHz.
(4) The CAS-induced change on tone detection thresholds was not correlated with the CAS-induced horizontal displacement of CEOAE or DPOAE I/O curves.
(5) The CAS effect on CEOAEs for a given click level was not correlated with the CAS effect on DPOAEs for a given *L*_2_.
(6) The horizontal displacements of CEOAEs and DPOAEs I/O curves induced by CAS tended to be correlated with each other, at least for conditions with the greater number of data points.
(7) The test–retest variability of the CAS effect was high overall but smaller for tone detection thresholds and CEOAEs than for DPOAEs.
(8) The weak correlations and poor reliability observed here could be related with inherent limitations of the study, such as the small range of clicks and *L*_2_ levels used, and/or with factors not related with the MOCR. Nonetheless, the present findings show that the different estimates of the MOCR strength cannot be used independently and assume that they provide similar results.

## Data Availability Statement

The raw data supporting the conclusions of this article will be made available by the authors, without undue reservation.

## Ethics Statement

The studies involving human participants were reviewed and approved by Comité de Bioética, Universidad de Salamanca. The participants provided their written informed consent to participate in this study.

## Author Contributions

MM-P performed the research, analyzed the data, and wrote the first draft of the manuscript. MM-P and EL-P edited the manuscript and wrote the manuscript. PJ provided technical tools. EL-P designed the research. All authors contributed to the article and approved the submitted version.

## Conflict of Interest

The authors declare that the research was conducted in the absence of any commercial or financial relationships that could be construed as a potential conflict of interest.

## Footnotes

1 CEOAEs were measured only over a 9-dB range because 51 dB pSPL was the lowest level at which participants showed valid OAEs (i.e., very few participants showed valid responses at lower levels) and 60 dB pSPL was the highest level we could use without large artifacts in our system using the linear mode of stimulation.

2 Other authors (e.g., Mishra and Lutman, 2013) have quantified the effect of CAS in percentage as follows: 100 × (OAE_*CTR*_-OAE_*CAS*_)/OAE_*CTR*_, with all OAE quantities in units of Pascal. We opted to calculate CAS suppression in dB instead of percentage because the two methods would lead to the same conclusions. This is because a given amount of suppression in dB always corresponds with the same amount of suppression in percentage, independent of the baseline (without CAS) response.
